# Expansion of Grocery Delivery and Access for Washington SNAP Participants During the COVID-19 Pandemic

**DOI:** 10.5888/pcd19.210412

**Published:** 2022-06-30

**Authors:** Shawna Beese, Ofer Amram, Acacia Corylus, Janessa M. Graves, Julie Postma, Pablo Monsivais

**Affiliations:** 1 College of Nursing, Washington State University, Spokane, Washington; 2 College of Agricultural, Human, and Natural Resource Sciences, SNAP-ED, Snohomish, Washington; 3 Elson S. Floyd College of Medicine, Washington State University, Spokane, Washington

**Figure:**
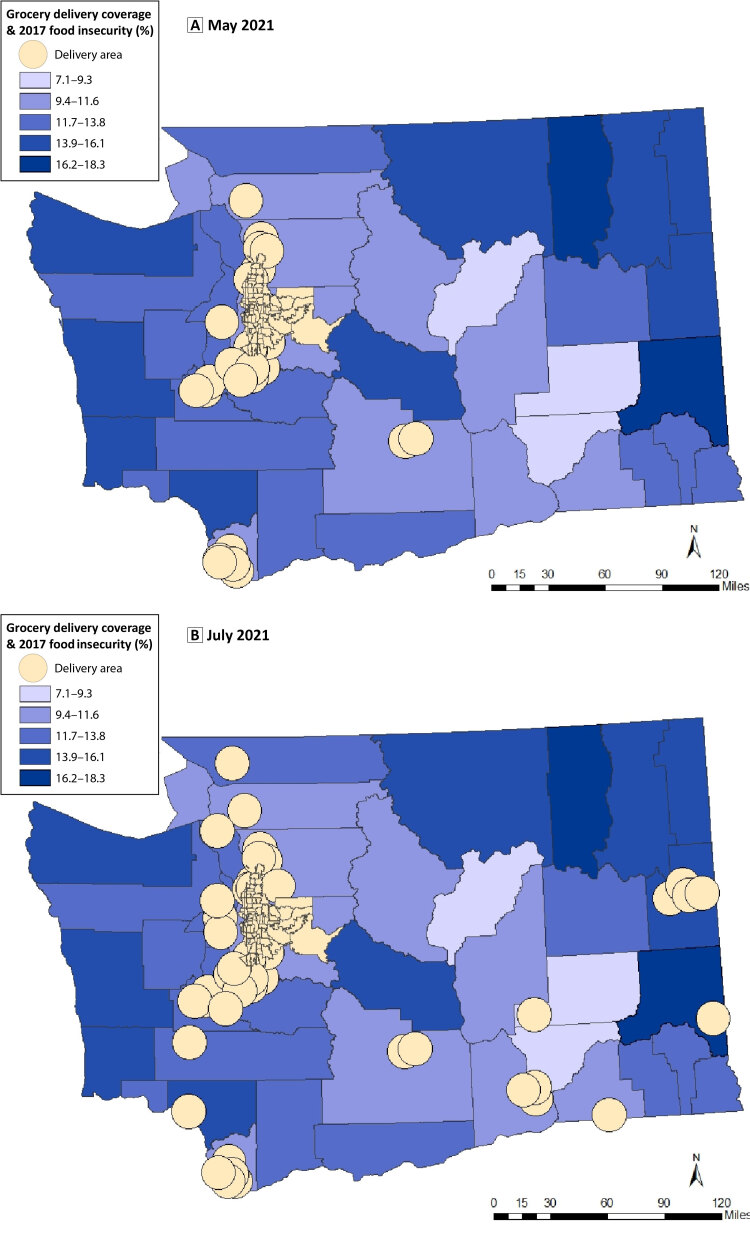
Online grocery delivery access for Washington State Supplemental Nutrition Assistance Program (SNAP) beneficiaries increased between May and July 2021, during the COVID-19 pandemic. The 2017 food insecurity rates spotlight the counties most vulnerable to food insecurity. Sources: Walmart delivery data, https://www.walmart.com/store/directory; Amazon delivery data, https://www.amazon.com; SNAP benefit data, https://www.ers.usda.gov/data-products/food-access-research-atlas/download-the-data; Washington 2010 census tract shapefile, https://ofm.wa.gov/washington-data-research/population-demographics/gis-data/census-geographic-files.

## Background

The COVID-19 pandemic ushered in an unprecedented food security crisis that will have long-term health effects (1). As society shut down to slow the spread of SARS-CoV-2, the interruptions tested the limits of our current food supply chain to meet supply and demand (2). Exigencies on our food system, particularly safety-net resources like food banks, increased by 50% nationwide (1). Inequities in access to healthy food became magnified, exacerbating disparities that existed before the pandemic, such as increased risk of food insecurity for people without college degrees and unemployed people (3).

The pandemic generated shifts toward home-based meal preparation and online grocery purchases with home delivery (4,5). State-level pilot programs for online grocery purchase and home delivery for Supplemental Nutrition Assistance Program (SNAP) beneficiaries were already mandated by the 2014 Farm Bill (6), but the pandemic accelerated these initiatives. Modernization of SNAP mirrors efforts spurred on by the pandemic, including national vendors’ investments in online grocery delivery infrastructure and expanded delivery services (2). Because of the rapid responses on behalf of private and governmental entities, evaluation of access to delivery services and beneficiary awareness of new online delivery services availability was needed.

The purpose of this GIS Snapshot was to present the results of a geospatial assessment of access to online grocery purchase and home delivery for SNAP beneficiaries in Washington State. A secondary aim of this study was to identify potentially vulnerable populations resulting from the current level of access.

## Data Sources and Map Logistics

We mapped the online grocery purchase and delivery coverage for SNAP recipients for Washington State in May 2021 and July 2021. Data sources included the GIS (geographic information system) census tract shapefile from the Washington Office of Financial Management (7). Census tract–level SNAP household data were obtained from the 2015 American Community Survey (ACS), accessed from the US Department of Agriculture Food Environment Atlas website (8).

The approved SNAP Electronic Benefits Transfer (EBT) delivery vendors were confirmed on the Food and Nutrition Service website in May 2021 and July 2021 (9). In July 2021, the 2 approved SNAP EBT online vendors were Amazon and Walmart delivery centers. One other retailer with a single location was also approved for online purchases, but this retailer did not have widespread delivery services, so its 1,530-person catchment was not included. The final analysis and reporting of the evaluation are at the county level.

We used ArcMAP Desktop version 10.8 (Esri) to overlay census tract data with the number of SNAP household units per census tract and the delivery areas of participating vendors. Each census tract was also identified as urban or rural; areas with populations under 2,500 people were classified rural and those with 2,500 people or more were classified urban (10). Delivery areas for Walmart were created with a 9-mile buffer around georeferenced Walmart distribution outlet points for Walmart. We selected a 9-mile radius based on Walmart’s delivery area, confirmed on their website in July 2021 and by telephone during confirmation calls to each store (11). The delivery area for Amazon Fresh was created using zip code polygons. All zip codes of the Amazon Fresh delivery services were confirmed on the Amazon Fresh website in July 2021 (12). The number of SNAP households was spatially analyzed by census tract population-weighted centroids within the defined delivery area boundaries.

We mapped the 2017 food insecurity rates by using a choropleth (shaded enumeration of prevalence rates) by county (13). Equal interval data breaks were used to categorize the food insecurity rates, expressed in percentages (13). The data sources for the 2017 food insecurity rates were from the Hunger in Washington website (14). The overall prevalence of food insecurity in Washington State was 11.5% and ranged from 7.1% (Franklin County) to 18.3% (Whitman County) (14).

## Highlights

According to 2015 American Community Survey data, 376,467 (11.8%) Washington households were receiving SNAP benefits. In July 2021, during our final analysis, 298,839 (79.4) households receiving SNAP benefits had access to online grocery purchases and delivery (Table). The number of Washington State residents receiving food assistance increased during the COVID-19 pandemic. According to Washington State Economic Services Administration reporting, the number of people on food assistance increased by 16% from September 2019 to July 2020, as reported through management accountability and performance statistics (Economic Services Administration, Management Accountability and Performance Statistics [ESA/EMAPS], the state SNAP client eligibility system).

**Table:** Washington State Supplemental Nutrition Assistance Program Online Grocery Delivery Access, May–July 2021

County	Number of households in county in 2015	Number of households with SNAP benefits in 2015	Number (%) of households with SNAP benefits with online delivery access
Adams	6,443	1,265	962 (76.0)
Asotin	9,989	1,782	—
Benton	73,896	10,176	8,992 (88.4)
Chelan	36,890	3,161	—
Clallam	36,526	4,860	—
Clark	175,854	25,515	24,652 (96.6)
Columbia	2,156	352	—
Cowlitz	44,178	8,908	7,280 (81.7)
Douglas	16,592	2,280	—
Ferry	4,485	728	—
Franklin	27,035	5,073	4,291 (84.6)
Garfield	1,245	95	—
Grant	36,385	6,434	—
Grays Harbor	35,816	5,901	—
Island	41,239	3,161	2,251 (71.2)
Jefferson	18,257	1,773	—
King	900,236	86,369	86,019 (99.6)
Kitsap	110,715	12,208	11,996 (98.3)
Kittitas	23,054	2,431	—
Klickitat	10,181	1,205	—
Lewis	34,759	6,373	3,381 (53.1)
Lincoln	5,962	484	—
Mason	33,172	4,103	—
Okanogan	22,901	3,328	—
Pacific	16,036	1,940	—
Pend Oreille	8,163	1,215	—
Pierce	339,501	44,959	43,334 (96.4)
San Juan	13,908	619	—
Skagit	52,846	7,445	5,439 (73.1)
Skamania	5,777	727	—
Snohomish	302,639	33,385	31,871 (95.5)
Spokane	210,709	33,984	31,890 (93.8)
Stevens	21,524	3,485	—
Thurston	113,750	12,767	11,656 (91.3)
Wahkiakum	2,118	322	—
Walla Walla	24,346	3,556	3,355 (94.3)
Whatcom	94,338	12,460	8,360 (67.1)
Whitman	20,381	1,844	1,252 (67.9)
Yakima	87,809	19,794	11,858 (59.9)
Washington State total	Not applicable	376,467	298,839 (79.4)

Abbreviation: —, no delivery available; SNAP, Supplemental Nutrition Assistance Program.

Sources:

Amazon. Amazon Fresh delivery [interactive database]. https://www.amazon.com. Accessed July 20, 2021.

Hunger in Washington. Food insecurity rate: by county. 2017. [interactive map]. https://www.livestories.com/statistics/hunger-in-washington/washington/food-insecurity. Accessed November 1, 2021.

US Census Bureau. National, state, and county housing unit totals: 2010–2019. Published April 20, 2022. Accessed April 27, 2022. https://www.census.gov/data/datasets/time-series/demo/popest/2010s-total-housing-units.html

US Department of Agriculture Food Atlas. 2015 Food Access Research Atlas published April 27, 2021. https://www.ers.usda.gov/data-products/food-access-research-atlas/download-the-data/. Accessed July 20, 2021.

Walmart. Walmart Store Directory [interactive databasehapefile]. https://ofm.wa.gov/washington-data-research/population-demographics/gis-data/census-geographic-files. Accessed November 1, 2021.

Washington Office of Financial Management. Washington 2010 Census County [shapefile]. https://ofm.wa.gov/washington-data-research/population-demographics/gis-data/census-geographic-files. Accessed November 1, 2021.

A preliminary survey conducted in May 2021 confirmed that most online grocery delivery access was concentrated in the Puget Sound region, the state’s largest metropolitan area. In July 2021, Walmart launched a delivery service expansion into other urban areas of the state. The expansion increased access from 169,507 (45.0%) of SNAP households statewide in May 2021 to 261,752 (69.5%) of SNAP households statewide. The July 2021 Walmart expansion substantially increased access to home delivery of groceries for Washington State by offering access to areas beyond the Puget Sound region and outlying communities of Vancouver and Yakima. In July 2021, administrative data from the ESA/EMAPS indicated that 2.3% of SNAP benefits were redeemed with online retailers.

Rural counties that were previously designated as food insecure continue to lack access to home delivery. Most households receiving SNAP were included in the new delivery coverage from Walmart’s expansion. However, Walmart’s expansion of online delivery services is concentrated in more densely populated areas of the state. The 2017 food insecurity rates indicate some of these counties without online delivery access, especially the northeastern Washington counties and the counties along the western coastline, are also food insecure (14).

## Action

Our analyses show the expansion of online grocery delivery serving SNAP recipients in Washington State. However, gaps in broadband coverage and lack of home computer technology may still serve as potential barriers to online purchases for rural populations in the state (15,16).

Using administrative data for geographic assessment to quantify access to online grocery delivery services was an essential step, but our analyses did not include assessment of other food assets such as food retail locations, food banks, pantries, and mobile markets (17,18). On the basis of the geographic assessment presented here, next steps include community-based approaches that allow for broader inventory of local food assets, which may be important for food access, particularly in communities with limited online grocery delivery. This involves asset-based inquiry to ground realities from rural food system leaders seeking innovative strategies to provide efficient and equitable solutions for food delivery.

Program administrators and municipal policy makers can use these maps to target underserved areas and strategize building partnerships with local vendors such as farmer’s co-ops and regional-based grocery outlets to fill the delivery needs for rural areas.
